# The Multiple Faces of Systemic Lupus Erythematosus: Pearls and Pitfalls for Diagnosis

**DOI:** 10.31138/mjr.130124.ppa

**Published:** 2024-06-30

**Authors:** Noemin Kapsala, Dionysis Nikolopoulos, Antonis Fanouriakis

**Affiliations:** 1 ”Attikon” University Hospital of Athens, Rheumatology and Clinical Immunology, Medical School, National and Kapodistrian University of Athens, Athens, Greece,; 2 Division of Rheumatology, Department of Medicine, Karolinska Institutet, Stockholm, Sweden

**Keywords:** systemic lupus erythematosus, diagnosis, criteria, clinical presentation

## Abstract

Systemic lupus erythematosus is the prototype multisystem autoimmune disorder characterised by a broad spectrum of organ involvement and a multitude of laboratory abnormalities. Clinical heterogeneity, unpredictable course and lack of pathognomonic clinical and serological features pose a considerable challenge in the diagnosis of SLE. The latter remains largely clinical, typically accompanied however by features of serologic autoimmunity, which are characteristic for the disease. Despite significant improvements in treatment strategies, an early diagnosis often continues to be an unmet need, as the median reported delay from symptom onset to SLE diagnosis is approximately 2 years. Classification criteria are usually used to support the diagnosis, yet with significant caveats. In this article, we provide an updated review of the clinical presentation of lupus and give clues for an accurate diagnosis.

## INTRODUCTION

Systemic lupus erythematosus (SLE) is a complex autoimmune disease with a broad spectrum of clinical manifestations and accompanying laboratory abnormalities.^1,2^ Its onset is often insidious, with clinically evident disease developing over years, following a period of preclinical evidence of autoimmunity.^3–5^ Early in the disease course, many patients present with only a few features of the disease, that can resemble other autoimmune, infectious, or haematological diseases.^6–11^ This paucity of specific manifestations, its insidious onset and the wide range of potential organ involvement often pose considerable challenges in the diagnosis of SLE.^3–5^

Although increasing awareness, mainly due to advances in autoantibodies and understanding of the disease presentation, has reduced the diagnostic delay from approximately 50 months before 1980 to approximately 24 months in current times, the time lag is still long.^3–5^ According to recent studies, the mean time between onset of symptoms and SLE diagnosis is approximately 2 years,^12–15^ although a longer time has been reported for males, childhood-onset, and late-onset SLE, probably due to the lower index of suspicion in these patients.^12,15–16^ The delay in SLE diagnosis may lead to respective delay in treatment initiation, thus increase the risk of organ damage and affect short- and long- term outcomes.^15–22^ Importantly, for patients with major organ involvement, such as nephritis or neuropsychiatric lupus, this delay in diagnosis and treatment initiation has been associated with adverse outcomes.^15,18–19,23^ Also, there is evidence to support that early initiation of glucocorticoids and immunosuppressive agents may lead to earlier disease remission, thus improving long-term prognosis.^18–23^ Although a universally accepted definition of what constitutes “early” diagnosis is lacking, and a window of opportunity for timely treatment has not been clearly defined, some studies suggest that patients diagnosed within 6 months from symptom onset experience lower rates of flares, hospitalisations, healthcare utilisation costs, and disease-related damage.^5,15–22^

## SLE CLASSIFICATION CRITERIA: CHRONICLE AND NEW DIAGNOSTIC TOOLS

The diagnosis of SLE remains clinical, typically supported by laboratory abnormalities indicative of autoimmune reactivity. In practice, since diagnostic criteria for the disease have not been developed, diagnosis is usually made in a patient who presents with a combination of specific clinical and immunologic features, following the exclusion of other diagnoses which can mimic SLE.^3–4,16,24–25^

The various sets of classification criteria of SLE reflect the multifaceted nature of the disease and they have been primarily developed to ensure the inclusion of homogeneous groups of patients in clinical and epidemiological studies.^26^ Although they are often used as a diagnostic aid in routine clinical practice, they cannot be applied in every individual case, since misclassification may occur due to their sensitivity and specificity (which are both less than 100%).^3–4,16,25,27–28^ Classification criteria with a high sensitivity may be used informally to support a clinical diagnosis, however, the possibility of ‘overdiagnosis’ is well recognised and should not be overlooked. ^3–4,16,25,27–29^ Following the first classification criteria from the American College of Rheumatology (ACR),^30^ the SLICC criteria, published in 2012, aimed for earlier diagnosis and higher sensitivity, by necessitating the patient to satisfy ≥4criteria (at least one clinical and one immunologic), or to have biopsy-proven lupus nephritis in the presence of antinuclear antibodies (ANA) or anti-double-stranded DNA antibodies.^31^ As compared with ACR-1997 criteria, the SLICC-2012 criteria demonstrated better sensitivity (94.6% vs 89.6%) with a similar or slightly lower specificity (95.5% vs 98.1%), ensuring classification of more patients at a population level.^32–39^

The most recent set of classification criteria have been published in 2019 following a combined effort from the European League Against Rheumatism (EULAR)/ACR, and requiring a positive ANA test at least once as entry criterion, combined with at least 10 additional points derived from weighted criteria, grouped in 7 clinical and 3 immunologic domains.^40^ Data from the validation cohort suggested that the EULAR/ACR-2019 criteria achieved a higher and similar sensitivity as compared with ACR 1997 (96.1% vs 82.8%) and SLICC 2012 (96.1% vs 96.7%), respectively.^40^ Accordingly, the specificity was similar to ACR 1997 criteria (93.4% vs 93.4%), but higher than SLICC 2012 (93.4% vs 83.7%).^40^ Following their publication, data from recent cohort studies and a meta-analysis showed that both the SLICC-2012 and EULAR/ACR-2019 have a higher sensitivity as compared with ACR-1997 criteria, and thus are more suitable for the identification of patients with early disease.^41–45^ The performance of existing sets of SLE classification criteria in several real-life patient cohorts is shown in **Table 1**.

**Table 1.** Performance of different SLE classification criteria in real-life settings.

	**ACR 97**		**SLICC 12**		**EULAR/ACR 19**	
*Cohort/dataset*	*Sensitivity*	*Specificity*	*Sensitivity*	*Specificity*	*Sensitivity*	*Specificity*
**EULAR/ACR 2019 validation cohort^46^**	83%	93%	97%	84%	96%	93%
**Linköping (Sweden)^43^**	83%	82%	100%	75%	93%	73%
**Crete (Greece)^41^**	86%	93%	91%	94%	89%	97%
**Beth Israel Deaconess (US)^47^**	93%	N/A	99%	N/A	95%	N/A
**SLICC 12 criteria scenarios^48^**	83%	96%	97%	84%	91%	89%
**Seoul (Korea)^49^**	96%	94%	99%	93%	98%	91%
**Shanghai (China)^50^**	75%	96%	92%	84%	96%	90%

SLE: Systemic Lupus Erythematosus; ACR: American College of Rheumatology; SLICC: Systemic Lupus International Collaborating Clinics; EULAR: European Alliance of Associations for Rheumatology; ACR: American College of Rheumatology; N/A: not applicable.

A diagnosis is often most challenging in the early stages of the disease, when the latter may manifest only a few or even a single clinical manifestation (organ-dominant lupus).^7,41,51^ In such settings, application of existing sets of classification criteria may assist diagnosis; in fact, the combination of all three sets ensures the highest sensitivity.^25,27,41^ Importantly, a recent retrospective study showed that different sets of criteria still fail to classify a significant proportion of cases at the time of diagnosis by an expert, and thus some patients with potentially high disease burden may be “missed”.^41^ This study proposed that modifications of classification criteria, such as the use of an alternative entry criterion in ANA-negative patients or the classification of patients with fewer criteria from multiple domains, may enhance their diagnostic accuracy, enabling an earlier diagnosis and timely treatment of more patients.^41^

Recently, Adamichou et al. introduced a clinically applicable model to assist SLE diagnosis, by the use of machine learning.^52^ The SLE Risk Probability Index (SLERPI) consists of 14 variably weighted clinical and serological SLE features, that can produce individualised risk probabilities for clinical SLE against competing rheumatologic diseases (i.e., “definite”, “likely/probable”, “possible”, “unlikely”), alike clinical diagnostic reasoning. In the binary model, a threshold >7 can be used as a dichotomous algorithm (i.e., SLE or not) with high accuracy (94.2%) for SLE diagnosis, including early and severe disease.^52^ By operating the full probabilistic model, the algorithm can be used to exclude SLE (risk probability <14%), confirm SLE (risk probability >86%), or suggest identification and monitoring of patients with intermediate probabilities (15–85%).^52^ Recent studies on SLERPI performance showed a similar or higher sensitivity [98.5% (95% CI; 96.7%–99.4%)] compared to the classification criteria sets, albeit with lower specificity [84.6% (95% CI; 76.9%–90.4%)].^53–54^ Interestingly, SLERPI showed a good diagnostic performance for the earlier identification of SLE, comparable with EULAR/ACR-2019 criteria, especially among patients with undifferentiated connective tissue disease and those with severe organ involvement, in an ambulatory basis, as well as in the hospital setting.^54–56^ Further research in cohorts with larger samples and diverse ethnic/racial characteristics, is needed to further establish the clinical utility and diagnostic value of this tool.

## CLINICAL PRESENTATION OF SLE

### How does lupus present?

A typical pattern of presenting clinical picture for lupus does not exist. Moreover, what makes the diagnosis often challenging, is the fact that the diverse array of manifestations often do not appear simultaneously but rather accumulate gradually over the disease course, occasionally with a time interval of several months or even years between them.^16,24,32^ Additionally, a considerable proportion of patients present with non-specific manifestations, such as arthritis, fever or fatigue, or with rare and atypical features, such as unusual rashes, neuropsychiatric manifestations, or abdominal vasculitis, among others. A small but challenging group of patients might present with single-organ involvement, the so called ‘organ-dominant lupus’.^4,25^ Sex, age and ethnicity may also influence the expression of SLE, as people of white race tend to develop milder phenotypes compared to Blacks or Asians.^4,12,16,57^

Mucocutaneous lesions and musculoskeletal involvement are almost universal in SLE, occurring in about 90% of SLE patients. Arthralgias and true synovitis are very common manifestations, with the majority of patients manifesting a symmetric non-erosive polyarthritis, usually early in the disease course.^4,32,57^

Lupus nephritis (LN) represents one of the most characteristic manifestations and usually, although not exclusively, occurs early in the disease course. Renal involvement presents in about 25–50% of patients, with higher percentages in certain ethnic groups, such as African-Americans.^32,57–62^

Hematologic involvement is also a frequent manifestation and can be a presenting manifestation of SLE. Its clinical presentation is highly variable, including the possible involvement of all three blood cell lines, while occasionally can be associated with severe lupus disease.^32,57, 63^ Notably, severe organ involvement in lupus has been associated with haematological abnormalities, with thrombocytopenia representing an independent risk factor of death and being associated with higher disease activity and greater damage in different SLE cohorts.^64–67^ Neuropsychiatric lupus (NPSLE) prevalence varies widely among different cohorts, owing to differences in definition and attribution to the disease *per se*. Importantly, most severe neuropsychiatric syndromes tend to manifest more frequently around the onset of SLE and are significantly more frequent as presenting manifestations in childhood-onset lupus.^68–72^

Importantly, non-infectious fever, recently included as a criterion in the 2019 EULAR/ACR criteria, with data from different early SLE cohorts, suggest the importance of this manifestation as an early feature, despite its obviously low specificity.^8,10^

## MULTISYSTEM VERSUS ORGAN-DOMINANT DISEASE

A minority of SLE patients may present with organ-dominant disease, often with a severe clinical manifestation.^25^ The diagnosis of organ-dominant disease is challenging, because these patients typically do not fulfil classification criteria; in these circumstances, judgment of a physician experienced in SLE may result in a diagnosis of ‘possible SLE’, only after rigorous exclusion of alternative causes for the patient’s symptoms. In such clinical scenarios, the diagnosis can be substantiated in patients displaying manifestations with a high specificity for SLE (e.g., malar rash, positivity for SLE-specific autoantibodies, such as anti-dsDNA or anti-Smith), or when there is histologic suspicion or confirmation of lupus, particularly in cases of nephritis and cutaneous lupus. In patients not falling into one of these categories, the diagnosis of SLE may be delayed until additional clinical or serological features accumulate, potentially affecting the long-term prognosis. For patients presenting with mild-moderate single-organ symptoms, a strategy of ‘watchful waiting’ or a therapeutic trial with low to moderate dose of glucocorticoids might be beneficial. By contrast, prompt interventions are needed for patients presenting with severe or life-threatening manifestations. In such situations, an aggressive work-up is warranted to rule out other life-threatening conditions, particularly those that can be exacerbated by immunosuppressive treatments like infections. Following this, with a possible diagnosis of SLE, immunosuppressive treatment is justified, under the supervision of an experienced SLE physician and multidisciplinary evaluation. Revisiting the accuracy of the diagnosis after a period of time in doubtful cases is of utmost importance.^73^

Contrary to organ-dominant presentation, diagnosing SLE in patients with multisystem involvement is generally considered as more straightforward, because these individuals usually have a more familiar clinical picture for physicians with less experience in the disease.^41^ Nonetheless, it is still important to consider various conditions that mimic SLE in the differential diagnosis, especially when specific features are absent. In cases with more benign clinical presentations, an optimal evaluation should rule out other connective tissue diseases and chronic infections, in the presence of suggestive symptoms. In patients with severe multi-system SLE requiring hospitalisation, it’s essential to consider viral infections, sepsis and full-blown vasculitis as potential alternative diagnoses that should be part of the corresponding diagnostic work-up. In this regard, the diagnosis of SLE always demands a holistic assessment that should be adapted to each individual case based on disease severity and available clinical and laboratory findings.

## SEX-AGE DIFFERENCES AND THE ROLE OF ETHNICITY

The incidence of childhood-onset SLE (cSLE, diagnosis in patients < 16 years old) among all SLE cases may account for up to 20% in different cohorts,^74^ while a similar incidence at 10–20% has been reported for SLE patients with late-onset disease (i.e. diagnosis after 50 years).^75^ Of note, most recent cohorts have reported a notable increase in the proportion of SLE patients with late diagnosis, and a higher average age at SLE diagnosis when compared to studies conducted prior to 2000.^75–77^ Traditionally, cSLE is considered a more severe form of lupus characterised by high disease activity, early accumulation of damage and increased need for potent immunosuppressive therapy.^74^ In a large cohort from the UK including patients with different ethnic backgrounds, cSLE was associated with a higher frequency of lupus nephritis, mucocutaneous manifestations, thrombocytopenia, haemolytic anaemia, seropositivity for SLE-specific antibodies, and with increased mortality rates, while adult-onset SLE patients were more like to exhibit arthritis and leukopenia.^78^ On the other hand, patients with late-onset SLE exhibit more frequently serositis and pulmonary involvement.^75,79^ Two recent studies comparing cSLE with late-onset disease showed that cSLE tend to manifest more frequently lupus nephritis, acute cutaneous lupus, alopecia, oral ulcers, and fever, while late-onset-SLE was linked to increased incidence of pleuritis and pericarditis.^58,80^

The characteristic 9–10:1 female-to-male ratio in SLE seems to diminish with increasing age at diagnosis, reaching as low as 2.6:1 for patients with late-onset disease and indicating that male patients tend to develop SLE at an older age.^75^ In a recent cohort study, male SLE patients had a higher mean age at disease onset and frequency of late-onset SLE.^81^ Numerous studies have sought to discern disparities between male and female SLE phenotypes, yielding conflicting findings. Nevertheless, a common trend in these studies suggests that male patients are more inclined to experience serositis, thrombosis, and late-onset disease.^82^ While earlier cohort studies reported a higher prevalence of renal involvement in males, recent reports do not confirm this observation; still, LN in men is associated with worse outcomes.^81–84^ Male SLE patients accumulate irreversible damage earlier in the disease course and have significantly higher SLICC damage index scores within the 5 first years of the disease, suggesting a more aggressive phenotype.^85^

Racial and ethnic background is an additional major determinant of the lupus phenotype. Collectively, most studies suggest a more severe disease in black, Hispanic, and Asian patients.^86^ Multi-ethnic cohort studies demonstrated that white patients exhibited the lowest prevalence of LN, while Asians are less likely to manifest neuropsychiatric events.^59^ Musculoskeletal and skin manifestations are commonly observed among white individuals, while chronic cutaneous lupus is more frequent among black SLE patients.^58,87^ Immune-mediated cytopenias are typical manifestations among patients of Asian origin, while serositis appears to be more prevalent in Hispanics.^86,88^ With respect to antibodies, Black and Asian individuals are more commonly positive for anti-Smith and anti-dsDNA, while antiphospholipid antibodies are more often positive in white SLE patients compared to other ethnicities.^86^

Based on all of the above, it becomes evident that physicians of multiple disciplines need to be well-informed about the varying SLE presenting pictures based on a patient’s sex, age and racial/ethnic background, in order to be able to tailor the diagnostic work-up for each individual patient.

## DIAGNOSIS OF LUPUS IN THE HOSPITAL SETTING

Although the majority of lupus patients are usually diagnosed in an outpatient setting, SLE can occasionally first present with severe or critical disease necessitating hospitalisation.^56,58^ Previous studies have reported significant variations in rates of hospitalisation and reasons of admission, mainly reflecting underlying socioeconomic and ethnic differences. Approximately 10–20% of SLE patients are hospitalised annually, the most common causes of admission being active disease (11%–80.8%) and infections (10.9%–37%).^89–97^ Among hospitalised SLE patients, about 20–30% are new cases diagnosed during hospitalisation.^56,92–97^

The clinical presentation of lupus flare in hospital setting varies among different studies and usually reflect severe forms of the disease. Lupus nephritis and haematological disorders are identified as the most frequent flare manifestations in most studies, accounting for 17.2%–63.4% and 17%–58%, respectively.^56,92–97^ We recently studied the clinical phenotypes of SLE patients diagnosed during hospitalisation and found that neuropsychiatric syndromes were the most common ones leading to admission, comprising 21% of patients (a generally higher proportion compared with previous literature, 1.6%–24.8%).^56^ Thus, NPSLE is an emerging severe lupus phenotype, given also the fact that most NPSLE events (30–40%) occur at disease onset or early in the disease course (within the first year).^51,58,70–72^ Thromboembolic events, serositis and musculoskeletal or constitutional manifestations were less common in hospitalised patients, possibly implying an outpatient management for these disease phenotypes.^56,92–97^ Importantly, while patients with SLE have an increased risk for cardiovascular events compared with the general population,^98–99^ these are less frequent reported flares, and mostly reflect lupus hospitalisations related to disease damage or comorbidities, rather than SLE itself.^56,92–97, 100^

## PRECLINICAL SLE, INCOMPLETE SLE AND UNDIFFERENTIATED CONNECTIVE TISSUE DISEASE

The period preceding a formal diagnosis of SLE has been referred to as “preclinical lupus”, “lupus-like disease” or “incomplete lupus” (ILE). Although there are no universally accepted definitions for these entities, in older literature ILE typically pertained to patients who had one or more, but fewer than four, ACR SLE classification criteria.^101^ Undifferentiated connective tissue disease (UCTD) is a broader term used for individuals who exhibit symptoms suggestive of a CTD but do not meet specific classification criteria for any.^102^ Among patients with UCTD, a small proportion (10–20%) will eventually develop SLE.^103^ To date, no single biomarker has been shown to predict the progression of these patients to definite SLE, thus, only close follow-up will promptly capture those individuals who transition to SLE. Female sex, younger age, malar rash, oral ulcers, and thrombocytopenia have been associated with transition from ILE to SLE in these studies, while ILE patients who remained stable over time were more likely to manifest arthritis and leukopenia.^104–107^ Importantly, ILE patients who transition to SLE during follow-up tend to exhibit a more benign disease course, characterised by a lower incidence of major organ involvement.^103^ Independent risk factors for transition from UCTD to SLE include fever, discoid lesions, serositis, anti-dsDNA and anti-Sm.^108–109^ Importantly, patients with either ILE or UCTD tend to progress to SLE within the first years following diagnosis, signifying the need for more tight monitoring during this early period.^110^

In contrast to incomplete/undifferentiated forms of lupus, preclinical SLE typically refers to individuals with serological evidence of autoimmunity (e.g., positivity for ANA and/or other autoantibodies), without any evident clinical symptoms. Several studies have been conducted to identify risk factors associated with the transition from the pre-clinical stage to definitive SLE, with the primary objective to minimise diagnostic delay. Approximately 90% of patients with SLE develop autoantibodies prior to diagnosis.^111^ Notably, non-specific antibodies including ANA, anti-phospholipid, anti-Ro, and anti-La, were detected at an earlier pre-clinical stage compared to SLE-specific antibodies, such as anti-Sm and anti-dsDNA antibodies.^111^ A Swedish SLE cohort study showed that 63% of SLE patients develop ANA approximately 5 years prior to the diagnosis, whereas anti-Sm antibodies become detectable closer to the time of diagnosis.^112^ Nevertheless, only a minority of SLE patients carry SLE-specific antibodies during the pre-clinical phase. Furthermore, non-specific antibodies, particularly ANA, are prevalent in a range of autoimmune disorders and can also be found in individuals without autoimmune conditions, rendering them unsuitable for diagnostic purposes. Results from the same Swedish cohort indicated that increased levels of IP-10 (interferon gamma induced protein 10) and inter-feron-a (IFN-a) are evident in SLE patients at pre-clinical stage.^113^ Indeed, recent evidence demonstrate that IFN-α might potentially serve as a surrogate marker for the transition from the pre-clinical stage to established SLE, and validation studies are currently in progress. In this regard, a higher IFN score along with family history could help to identify ANA-positive individuals who will progress to definitive connective tissue disease, including SLE, although IFN upregulation (albeit lower) was evident even in ANA-positive individuals who did not ultimately progress to SLE.^114^

Ongoing research in the field of preclinical disease is of utmost importance for uncovering diagnostic biomarkers to identify individuals at high-risk for closer monitoring and intervention, ideally aiming at preventing the transition to full-blown disease.

## References

[1] Tsokos G. Systemic lupus erythematosus. N Engl J Med 2011;365(22):2110–21. 22129255 10.1056/NEJMra1100359

[2] Agmon-Levin N Mosca M Petric M Shoenfeldad Y. Systemic lupus erythematosus one disease or many? Autoimmun Rev 2012;11:593–595. 22041578 10.1016/j.autrev.2011.10.020

[3] Doria A Zen M Canova M Bettio S Bassi N Nalotto L SLE diagnosis and treatment: when early is early. Autoimmun Rev 2010; 10(1):55–60. 20813207 10.1016/j.autrev.2010.08.014

[4] Bertsias G Pamfil C Fanouriakis A Boumpas D. Diagnostic criteria for systemic lupus erythematosus: has the time come? Nat Rev Rheumatol 2013; 9(11):687–94. 23838616 10.1038/nrrheum.2013.103

[5] Piga M Arnaud L. The Main Challenges in Systemic Lupus Erythematosus: Where Do We Stand? J Clin Med 2021;10(2):243. 33440874 10.3390/jcm10020243 PMC7827672

[6] Gatto M Saccon F Zen M Iaccarino L Doria A. Preclinical and early systemic lupus erythematosus. Best Pract Res Clin Rheumatol 2019;33(4):101422. 31810542 10.1016/j.berh.2019.06.004

[7] Heinlen L McClain M Merrill J Akbarali Y Edgerton C Harley J Clinical criteria for systemic lupus erythematosus precede diagnosis, and associated autoantibodies are present before clinical symptoms. Arthritis Rheum 2007;56(7):2344–51. 17599763 10.1002/art.22665

[8] Mosca M Costenbader K Johnson S Lorenzoni V Sebastiani GD Hoyer B Brief report: how do patients with newly diagnosed systemic lupus erythematosus present? A multicenter cohort of early systemic lupus erythematosus to inform the development of new classification criteria. Arthritis Rheum 2019;71: 91–98. 10.1002/art.40674 30035365

[9] Rees F Doherty M Lanyon P Davenport G Riley R Zhang W Early Clinical Features in Systemic Lupus Erythematosus: Can They Be Used to Achieve Earlier Diagnosis? A Risk Prediction Model. Arthritis Care Res (Hoboken) 2017;69(6):833–841. 27588834 10.1002/acr.23021

[10] Leuchten N Milke B Winkler-Rohlfing B Daikh D Dörner T Johnson S Early symptoms of systemic lupus erythematosus (SLE) recalled by 339 SLE patients. Lupus 2018;27(9):1431–6. 29771193 10.1177/0961203318776093

[11] Sebastiani GD Prevete I Piga M Iuliano A Bettio S Bortoluzzi A Early Lupus Project – A multicentre Italian study on systemic lupus erythematosus of recent onset. Lupus 2015;24(12):1276–82. 25979916 10.1177/0961203315585817

[12] Nightingale A Davidson J Molta C Kan H McHugh N. Presentation of SLE in UK primary care using the Clinical Practice Research Datalink. Lupus Sci Med 2017;4:e000172. 28243454 10.1136/lupus-2016-000172 PMC5307373

[13] Morgan C Bland A Maker C Dunnage J Bruce I. Individuals living with lupus: findings from the LUPUS UK Members Survey 2014. Lupus 2018;27(4):681–687. 29310537 10.1177/0961203317749746 PMC5888773

[14] Ozbek S Sert M Paydas S Soy M. Delay in the diagnosis of SLE: the importance of arthritis/arthralgia as the initial symptom. Acta Med Okayama 2003;57(4):187–90. 14627070 10.18926/AMO/32807

[15] Kapsala N Nikolopoulos D Flouda S Chavatza A Tseronis D Aggelakos M From first symptoms to diagnosis of systemic lupus erythematosus: mapping the journey of patients in an observational study. Clin Exp Rheumatol 2023;41(1):74–81. 35485411 10.55563/clinexprheumatol/x3s9td

[16] Gergianaki I Bertsias G. Systemic Lupus Erythematosus in Primary Care: An Update and Practical Messages for the General Practitioner. Front Med (Lausanne) 2018;5:161. 29896474 10.3389/fmed.2018.00161 PMC5986957

[17] Kernder A Richter J Fischer-Betz R Winkler-Rohlfing B Brinks R Aringer M Delayed diagnosis adversely affects outcome in systemic lupus erythematosus: Cross sectional analysis of the LuLa cohort. Lupus 2021;30(3) 431–438. 33402036 10.1177/0961203320983445 PMC7933718

[18] Esdaile J Joseph L MacKenzie T Kashgarian M Hayslett J. The benefit of early treatment with immunosuppressive agents in lupus nephritis. J Rheumatol 1994;21(11): 2046–51. 7869308

[19] Faurschou M Starklint H Halberg P Jacobsen S. Prognostic factors in lupus nephritis: diagnostic and therapeutic delay increases the risk of terminal renal failure. J Rheumatol 2006;33(8):1563–9. 16881113

[20] Doria A Iaccarino L Ghirardello A Zampieri A Arienti S Sarzi-Puttini P Long-term prognosis and causes of death in systemic lupus erythematosus. Am J Med 2006;119(8):700–6. 16887417 10.1016/j.amjmed.2005.11.034

[21] Nossent J Kiss E Rozman B Pokorny G Vlachoyiannopoulos P Olesinska M Disease activity and damage accrual during the early disease course in a multinational inception cohort of patients with systemic lupus erythematosus. Lupus 2010;19:949–56. 20375124 10.1177/0961203310366572

[22] Oglesby A Korves C Laliberte F Dennis G Rao S Suthoff ED Impact of early versus late systemic lupus erythematosus diagnosis on clinical and economic outcomes. Appl Health Econ Health Policy 2014;12:179–90. 24573911 10.1007/s40258-014-0085-x

[23] Lu X Gu Y Wang Y Chen S Ye S. Prognostic factors of lupus myelopathy. Lupus 2008;17(4):323–8. 18413414 10.1177/0961203307088005

[24] Maddison J. Is it SLE? Best Pract Res Clin Rheumatol 2002;16(2):167–80. 12041947 10.1053/berh.2001.0219

[25] Fanouriakis A Tziolos N Bertsias G Boumpas D. Update on the diagnosis and management of systemic lupus erythematosus. Ann Rheum Dis 2021;80(1):14–25. 33051219 10.1136/annrheumdis-2020-218272

[26] Aggarwal R Ringold S Khanna D Neogi T Johnson S Miller A Distinctions Between Diagnostic and Classification Criteria? Arthritis Care Res (Hoboken) 2015;67(7):891–7. 25776731 10.1002/acr.22583 PMC4482786

[27] Aringer M Petrib M. New Classification Criteria for SLE. Curr Opin Rheumatol 2020;32(6):590–6. 32925250 10.1097/BOR.0000000000000740 PMC8020886

[28] Aringer M Costenbader K Dörner T Johnson S. Advances in SLE classification criteria. J Autoimmun 2022;132:102845. 35725680 10.1016/j.jaut.2022.102845

[29] Hawkes C Giovannoni G Lechner-Scott J Levy M Pohl D.. Is multiple sclerosis overdiagnosed? Mult Scler Relat Disord 2021:47:102721–7. 33602498 10.1016/j.msard.2020.102721

[30] Hochberg M. Updating the American College of rheumatology revised criteria for the classification of systemic lupus erythematosus. Arthritis Rheum 1997;40(9):1725. 10.1002/art.1780400928 9324032

[31] Petri M Orbai AM Alarcón G Gordon C Merrill J Fortin P Derivation and validation of the systemic lupus international collaborating clinics classification criteria for systemic lupus erythematosus. Arthritis Rheum 2012;64:2677–86. 22553077 10.1002/art.34473 PMC3409311

[32] Huang X Zhang Q Zhang H Lu Q. A Contemporary Update on the Diagnosis of Systemic Lupus Erythematosus. Clin Rev Allergy Immunol 2022; 63(3):311–329. 35064901 10.1007/s12016-021-08917-7

[33] Hartman E van Royen-Kerkhof A Jacobs J Welsing P Fritsch-Stork R. Performance of the 2012 Systemic Lupus International Collaborating Clinics classification criteria versus the 1997 American College of Rheumatology classification criteria in adult and juvenile systemic lupus erythematosus. A systematic review and meta-analysis. Autoimmun Rev 2018;17(3):316–322. 29366725 10.1016/j.autrev.2018.01.007

[34] Tao J Hiraki L Levy D Silverman E. Comparison of sensitivities of American College of Rheumatology and Systemic Lupus International Collaborating Clinics Classification Criteria in childhood-onset systemic lupus erythematosus. J Rheumatol 2019;46(7):731–8. 30770510 10.3899/jrheum.180337

[35] Fonseca A Gaspar-Elsas MI Land M de Oliveira S. Comparison between three systems of classification criteria in juvenile systemic lupus Erythematous. Rheumatology (Oxford) 2015;54(2):241–7. 25125590 10.1093/rheumatology/keu278

[36] Ighe A Dahlström Ö Skogh T Sjöwall C. Application of the 2012 systemic lupus international collaborating clinics classification criteria to patients in a regional Swedish systemic lupus erythematosus register. Arthritis Res Ther 2015;17(1):3. 25575961 10.1186/s13075-015-0521-9 PMC4318183

[37] Inês L Silva C Galindo M López-Longo F Terroso G Romão V Classification of systemic lupus erythematosus: systemic lupus international collaborating clinics versus American College of rheumatology criteria. A comparative study of 2,055 patients from a reallife, International systemic lupus erythematosus cohort. Arthritis Care Res (Hoboken) 2015;67(8):1180–5. 25581417 10.1002/acr.22539

[38] Izmirly P Wan I Sahl S Buyon J Belmont H Salmon J The incidence and prevalence of systemic lupus erythematosus in New York County (Manhattan), New York: the Manhattan lupus surveillance program. Arthritis Rheumatol 2017;69(10):2006–17. 28891252 10.1002/art.40192 PMC11102806

[39] Ungprasert P Sagar V Crowson C Amin S Makol A Ernste F Incidence of systemic lupus erythematosus in a population-based cohort using revised 1997 American College of rheumatology and the 2012 systemic lupus international collaborating clinics classification criteria. Lupus 2017;26(3):240–7. 27365370 10.1177/0961203316657434 PMC5201452

[40] Aringer M Costenbader K Daikh D Brinks R Mosca M Ramsey-Goldman R 2019 European League against Rheumatism/American College of rheumatology classification criteria for systemic lupus erythematosus. Arthritis Rheumatol 2019;71(9):1400–12. 31385462 10.1002/art.40930 PMC6827566

[41] Adamichou C Nikolopoulos D Genitsaridi I Bortoluzzi A Fanouriakis A Papastefanakis E In an early SLE cohort the ACR-1997, SLICC-2012 and EULAR/ACR-2019 criteria classify non-overlapping groups of patients: use of all three criteria ensures optimal capture for clinical studies while their modification earlier classification and treatment. Ann Rheum Dis 2020;79(2):232–41. 31704720 10.1136/annrheumdis-2019-216155

[42] Lu W Tian F Ma J Zhong Y Liu Z Xue L.. Diagnostic accuracy of the European League against rheumatism/American College of Rheumatology-2019 versus the Systemic Lupus International Collaborating Clinics-2012 versus the ACR-1997 classification criteria in adult systemic lupus erythematosus: A systematic review and meta-analysis. Front Immunol 2022:13:1023451. 36311745 10.3389/fimmu.2022.1023451 PMC9599400

[43] Dahlström Ö Sjöwall C. The diagnostic accuracies of the 2012 SLICC criteria and the proposed EULAR/ACR criteria for systemic lupus erythematosus classification are comparable. Lupus 2019;28(6):778–82. 31046572 10.1177/0961203319846388 PMC6537028

[44] Batu E Akca U Kısaarslan A Sağ E Demir F Demir S The performances of the ACR 1997, SLICC 2012, and EULAR/ACR 2019 classification criteria in pediatric systemic lupus erythematosus. J Rheumatol 2021;48(6):907–14. 33191281 10.3899/jrheum.200871

[45] Chang L Huang P Kuo H Tu YK Tseng PT Liang CS Diagnostic accuracy of the American College of Rheumatology-1997, the Systemic Lupus International Collaborating Clinics-2012, and the European League Against Rheumatism-2019 criteria for juvenile systemic lupus erythematosus: A systematic review and network meta-analysis. Autoimmun Rev 2022;21(9):103144. 35842200 10.1016/j.autrev.2022.103144

[46] Johnson SR Brinks R Costenbader KH Daikh D Mosca M Ramsey-Goldman R Performance of the 2019 EULAR/ACR classification criteria for systemic lupus erythematosus in early disease, across sexes and ethnicities. Ann Rheum Dis 2020 Oct;79(10):1333–1339. 32816709 10.1136/annrheumdis-2020-217162

[47] Rubio J Krishfield S Kyttaris VC. Application of the 2019 European League Against Rheumatism/American College of Rheumatology systemic lupus erythematosus classification criteria in clinical practice: a single center experience. Lupus 2020 Apr;29(4):421–425. 32098572 10.1177/0961203320908939

[48] Petri M Goldman DW Alarcón GS Gordon C Merrill JT Fortin PR Comparison of the 2019 European Alliance of Associations for Rheumatology/American College of Rheumatology Systemic Lupus Erythematosus Classification Criteria With Two Sets of Earlier Systemic Lupus Erythematosus Classification Criteria. Arthritis Care Res (Hoboken) 2021 Sep;73(9):1231–5. 32433832 10.1002/acr.24263 PMC10711744

[49] Lee EE Lee EB Park JK Lee EY Song YW. Performance of the 2019 European League Against Rheumatism/American College of Rheumatology classification criteria for systemic lupus erythematosus in Asian patients: a single-centre retrospective cohort study in Korea. Clin Exp Rheumatol 2020 Nov–Dec;38(6):1075–9. 32083550

[50] Teng J Ye J Zhou Z Lu C Chi H Cheng X A comparison of the performance of the 2019 European League Against Rheumatism/American College of Rheumatology criteria and the 2012 Systemic Lupus International Collaborating Clinics criteria with the 1997 American College of Rheumatology classification criteria for systemic lupus erythematous in new-onset Chinese patients. Lupus 2020 May;29(6):617–24. 32216517 10.1177/0961203320914356

[51] Gergianaki I Fanouriakis A Repa A Tzanakakis M Adamichou C Pompieri A Epidemiology and burden of systemic lupus erythematosus in a Southern European population: data from the community-based lupus registry of Crete, Greece. Ann Rheum Dis 2017;76(12):1992–2000. 28780511 10.1136/annrheumdis-2017-211206

[52] Adamichou C Genitsaridi I Nikolopoulos D Nikoloudaki M Repa A Bortoluzzi A Lupus or not? SLE Risk Probability Index (SLERPI): a simple, clinician-friendly machine learning-based model to assist the diagnosis of systemic lupus erythematosus. Ann Rheum Dis 2021;80(6):758–66. 33568388 10.1136/annrheumdis-2020-219069 PMC8142436

[53] Tan B Tang I Bonin J Koelmeyer R Hoi A. The performance of different classification criteria for systemic lupus erythematosus in a real-world rheumatology department. Rheumatology (Oxford) 2022;61(11):4509–13. 35348630 10.1093/rheumatology/keac120 PMC9629341

[54] Zhang L Lu W Yan D Liu Z Xue L. Systemic Lupus Erythematosus Risk Probability Index: ready for routine use? Results from a Chinese cohort. Lupus Sci Med. 2023;10:e000988. 37696613 10.1136/lupus-2023-000988 PMC10496657

[55] Erden A Apaydın H Fanouriakis A Güven SC Armagan B Akyüz Dağlı P Performance of the systemic lupus erythematosus risk probability index in a cohort of undifferentiated connective tissue disease. Rheumatology (Oxford) 2022;61(9):3606–3613. 35015853 10.1093/rheumatology/keac005

[56] Kapsala N Nikolopoulos D Flouda S Chavatza A Tseronis D Aggelakos M First Diagnosis of Systemic Lupus Erythematosus in Hospitalised Patients: Clinical Phenotypes and Pitfalls for the Non-Specialist Am J Med 2022;135(2):244–253.e3. 34411524 10.1016/j.amjmed.2021.07.015

[57] Kado R. Systemic Lupus Erythematosus for Primary Care. Prim Care 2018;45(2):257–70. 29759123 10.1016/j.pop.2018.02.011

[58] Nikolopoulos D Kostopoulou M Pieta A Karageorgas T Tseronis D Chavatza K Evolving phenotype of systemic lupus erythematosus in Caucasians: low incidence of lupus nephritis, high burden of neuropsychiatric disease and increased rates of late-onset lupus in the ‘Attikon’ cohort. Lupus 2020 Apr; 29(5):514–22. 32106788 10.1177/0961203320908932 PMC7168806

[59] Anders HJ Saxena R Zhao MH Parodis I Salmon JE Mohan C. Lupus nephritis. Nat Rev Dis Primers 2020;23;6(1):7. 31974366 10.1038/s41572-019-0141-9

[60] Hanly JG O’Keeffe AG Su L Urowitz MB Romero-Diaz J Gordon C The frequency and outcome of lupus nephritis: results from an international inception cohort study. Rheumatol (Oxford) 2015;55:252–62. 10.1093/rheumatology/kev311 PMC4939728 26342222

[61] Tesar V Hruskova Z. Lupus Nephritis: A Different Disease in European Patients? Kidney Dis (Basel) 2015;1(2):110–8. 27536671 10.1159/000438844 PMC4934820

[62] Patel M Clarke A I Bruce I Symmons D. The prevalence and incidence of biopsy-proven lupus nephritis in the UK: Evidence of an ethnic gradient. Arthritis Rheum 2006;54(9):2963–9. 16947632 10.1002/art.22079

[63] Fayyaz A Igoe A Kurien BT Danda D James JA Stafford HA Haematological manifestations of lupus. Lupus Sci Med 2015;2(1):e000078. 25861458 10.1136/lupus-2014-000078 PMC4378375

[64] Zhu FX Huang JY Ye Z Wen QQ Wei JC. Risk of systemic lupus erythematosus in patients with idiopathic thrombocytopenic purpura: a population-based cohort study. Ann Rheum Dis 2020;79(6):793–9. 32241798 10.1136/annrheumdis-2020-217013

[65] Zhao H Li S Yang R. Thrombocytopenia in patients with systemic lupus erythematosus: significant in the clinical implication and prognosis. Platelets 2010;21(5):380–5. 20433308 10.3109/09537101003735564

[66] Ziakas PD Giannouli S Zintzaras E Tzioufas AG Voulgarelis M. Lupus thrombocytopenia: clinical implications and prognostic significance. Ann Rheum Dis 2005;64:1366–1369. 16100344 10.1136/ard.2004.033100 PMC1755663

[67] Fanouriakis A Bertsias G Boumpas D. Population-based studies in systemic lupus erythematosus: immune thrombocytopenic purpura or ‘blood-dominant’ lupus? Ann Rheum Dis 2020;79(6):683–4. 32312772 10.1136/annrheumdis-2020-217356

[68] Kivity S Agmon-Levin N Zandman-Goddard G Chapman J Shoenfeld Y. Neuropsychiatric lupus: a mosaic of clinical presentations. BMC Med 2015;4;13:43. 25858312 10.1186/s12916-015-0269-8 PMC4349748

[69] Nikolopoulos D Fanouriakis A Boumpas D. Cerebrovascular Events in Systemic Lupus Erythematosus: Diagnosis and Management. Mediterr J Rheumatol 2019;30(1): 7–15. 32185337 10.31138/mjr.30.1.7 PMC7045913

[70] Sarwar S Mohamed AS Rogers S Sarmast ST Kataria S Mohamed KH Neuropsychiatric Systemic Lupus Erythematosus: A 2021 Update on Diagnosis, Management, and Current Challenges. Cureus 2021;13(9):e17969. 34667659 10.7759/cureus.17969 PMC8516357

[71] Carrión-Barberà I Salman-Monte T Vílchez-Oya F Monfort J. Neuropsychiatric involvement in systemic lupus erythematosus: A review. Autoimmun Rev 2021;20(4):102780. 33609799 10.1016/j.autrev.2021.102780

[72] Bertsias GK Ioannidis JP Aringer M Bollen E Bombardieri S Bruce IN EULAR recommendations for the management of systemic lupus erythematosus with neuropsychiatric manifestations: report of a task force of the EULAR standing committee for clinical affairs. Ann Rheum Dis 2010;69(12):2074–82. 20724309 10.1136/ard.2010.130476

[73] Magro-Checa C Zirkzee EJ Beaart-van de Voorde LJJ Middelkoop HA van der Wee NJ Huisman MV Value of multidisciplinary reassessment in attribution of neuropsychiatric events to systemic lupus erythematosus: prospective data from the Leiden NPSLE cohort. Rheumatology (Oxford) 2017;56(10):1676–83. 28339952 10.1093/rheumatology/kex019

[74] Smith EMD Lythgoe H Midgley A Beresford MW Hedrich CM. Juvenile-onset systemic lupus erythematosus: Update on clinical presentation, pathophysiology and treatment options. Clin Immunol 2019;209:108274. 31678365 10.1016/j.clim.2019.108274

[75] Arnaud L Mathian A Boddaert J Amoura Z. Late-onset systemic lupus erythematosus: epidemiology, diagnosis and treatment. Drugs Aging 2019;29(3):181–9. 10.2165/11598550-000000000-00000 22263748

[76] Arbuckle MR McClain MT Rubertone MV Scofield RH Dennis GJ James JA Development of autoantibodies before the clinical onset of systemic lupus erythematosus. N Engl J Med 2003;349(16):1526–33. 14561795 10.1056/NEJMoa021933

[77] Nikolopoulos DS Kostopoulou M Pieta A Flouda S Chavatza K Banos A Transition to severe phenotype in systemic lupus erythematosus initially presenting with non-severe disease: implications for the management of early disease. Lup Sci Med 2020;7:e000394. 10.1136/lupus-2020-000394 PMC7326262 32601172

[78] Ambrose N Morgan TA Galloway J Ionnoau Y Beresford MW Isenberg DA; UK JSLE Study Group Differences in disease phenotype and severity in SLE across age groups. Lupus 2016;25(14):1542–50. 27147622 10.1177/0961203316644333 PMC5089221

[79] Boddaert J Huong DLT Amoura Z Wechsler B Godeau P Piette JC. Late-onset systemic lupus erythematosus: a personal series of 47 patients and pooled analysis of 714 cases in the literature. Medicine (Baltimore) 2004;83(6):348–59. 15525847 10.1097/01.md.0000147737.57861.7c

[80] Mongkolchaiarunya J Wongthanee A Kasitanon N Louthrenoo W. Comparison of clinical features, disease activity, treatment and outcomes between late-onset and early-onset patients with systemic lupus erythematosus. A sex- and year at diagnosis-matched controlled study. Adv Rheumatol 2023;63(1):20. 37127712 10.1186/s42358-023-00297-0

[81] Trentin F Signorini V Manca ML Cascarano G Gualtieri L Schilirò D Gender differences in SLE: report from a cohort of 417 Caucasian patients. Lupus Sci Med 2023;10(1):e000880. 37185240 10.1136/lupus-2022-000880 PMC10151995

[82] Tan TC Fang H Magder LS Petri MA. Differences between male and female systemic lupus erythematosus in a multiethnic population. J Rheumatol 2012;39(4):759–69. 22382348 10.3899/jrheum.111061 PMC3605704

[83] Ward MM Studenski S. Systemic lupus erythematosus in men: a multivariate analysis of gender differences in clinical manifestations. J Rheumatol 1990;17(2):220–4. 2319521

[84] Specker C Becker A Lakomek HJ Bach D Grabensee B. Systemischer Lupus Erythematodes bei Männern--Eine andere Krankheitsprognose? [Systemic lupus erythematosus in men--a different prognosis?]. Z Rheumatol 1994;53(6):339–45. 7871906

[85] Andrade RM Alarcón GS Fernández M Apte M Vilá LM Reveille JD Accelerated damage accrual among men with systemic lupus erythematosus: XLIV. Results from a multiethnic US cohort. Arthritis Rheum 2007;56(2):622–30. 17265497 10.1002/art.22375

[86] Ugarte-Gil MF Pons-Estel GJ Harvey GB Vilá LM Griffin R Alarcón GS. Applying the 2019 European Alliance of Associations for Rheumatology/American College of Rheumatology Lupus Criteria to Patients From the LUMINA Cohort: Results From the Multiethnic, Multicenter US Cohort. Arthritis Care Res (Hoboken) 2021;73(10):1451–1455. 32598552 10.1002/acr.24367

[87] Vilá LM Alarcón GS McGwin G Jr Friedman AW Baethge BA Bastian HM Early clinical manifestations, disease activity and damage of systemic lupus erythematosus among two distinct US Hispanic subpopulations. Rheumatology (Oxford) 2004;43(3):358–63. 14623949 10.1093/rheumatology/keh048

[88] Suda M Kishimoto M Ohde S Okada M. Validation of the 2019 ACR/EULAR classification criteria of systemic lupus erythematosus in 100 Japanese patients: a real-world setting analysis. Clin Rheumatol 2020;39(6):1823–7. 32052228 10.1007/s10067-019-04848-z

[89] Pires da Rosa G Cervera R Espinosa G. Causes of Hospitalisation in Systemic Lupus Erythematosus: A Narrative Review. Curr Rheumatol Rev 2021;17(1):29–40. 32718295 10.2174/1573397116666200727145818

[90] Edwards CJ Lian TY Badsha H Teh CL Arden N Chng HH. Hospitalisation of individuals with systemic lupus erythematosus: characteristics and predictors of outcome. Lupus 2003;12(9):672–6. 14514129 10.1191/0961203303lu452oa

[91] Somaily M Asiri S Aseery L Asiri B Gammash N Alhumayed R Causes and Outcomes of Hospitalisation among Systemic Lupus Erythematosus Patients in Aseer Central Hospital, Saudi Arabia: A Retrospective Study. Egypt J Hosp Med 2018;71(1):2358–2364.

[92] Lee J Dhillon N Pope J. All-cause hospitalisations in systemic lupus erythematosus from a large Canadian referral centre. Rheumatology 2013;52:905909; 10.1093/rheumatology/kes391 23307831

[93] Shoeib SA Elhamid AE Elshebiny EM Shehata YA Elshormilisy AA Mazen MR. Hospitalisation of systemic lupus patients: Causes and outcomes. Menoufia Med J 2017;30: 1210–1213.

[94] Jallouli M Hriz H Cherif Y Marzouk S Snoussi M Frikha F Causes and outcome of hospitalisations in Tunisian patients with systemic lupus erythematosus. Lupus Sci Med 2014;1(1): e000017. 25396063 10.1136/lupus-2014-000017 PMC4225742

[95] Selvananda S Chong YY Thundyil RJ. Disease activity and damage in hospitalised lupus patients: a Sabah perspective. Lupus 2020;29(3):344–50. 32046576 10.1177/0961203320904155

[96] Busch RW Kay SD Voss A. Hospitalisations among Danish SLE patients: a prospective study on incidence, causes of admission and risk factors in a population-based cohort. Lupus 2018;27(1):165–71. 29050537 10.1177/0961203317734919

[97] Teh CL Ling GR. Causes and predictors of mortality in hospitalised lupus patient in Sarawak General Hospital, Malaysia. Lupus 2013;22(1):106–11. 23112253 10.1177/0961203312465780

[98] Schattner A Liang H. The cardiovascular burden of lupus: a complex challenge. Arch Intern Med 2003;163(13):1507–10. 12860571 10.1001/archinte.163.13.1507

[99] Karrar A Sequeira W Block JA. Coronary artery disease in systemic lupus erythematosus: A review of the literature. Semin Arthritis Rheum 2001;30(6):436–43. 11404827 10.1053/sarh.2001.23498

[100] Thorburn CM Ward MM. Hospitalisations for coronary artery disease among patients with systemic lupus erythematosus. Arthritis Rheum 2003;48(9):2519–23. 13130471 10.1002/art.11241

[101] Greer JM Panush RS. Incomplete lupus erythematosus. Arch Intern Med 1989;149(11):2473–6. 2818107

[102] Alarcón GS Williams GV Singer JZ Steen VD Clegg DO Paulus HE Early undifferentiated connective tissue disease. I. Early clinical manifestation in a large cohort of patients with undifferentiated connective tissue diseases compared with cohorts of well established connective tissue disease. J Rheumatol 1991;18(9):1332–9. 1757934

[103] Sparks JA Costenbader KH. Genetics, environment, and gene-environment interactions in the development of systemic rheumatic diseases. Rheum Dis Clin North Am 2014;40(4):637–57. 25437282 10.1016/j.rdc.2014.07.005 PMC4250576

[104] Olsen NJ Li QZ Quan J Wang L Mutwally A Karp DR. Autoantibody profiling to follow evolution of lupus syndromes. Arthritis Res Ther 2012;14(4):R174. 22838636 10.1186/ar3927 PMC3580568

[105] Vilá LM Mayor AM Valentín AH García-Soberal M Vilá S. Clinical outcome and predictors of disease evolution in patients with incomplete lupus erythematosus. Lupus 2000;9(2):110–5. 10787007 10.1191/096120300678828073

[106] Swaak AJ van de Brink H Smeenk RJ Manger K Kalden JR Tosi S Incomplete lupus erythematosus: results of a multicentre study under the supervision of the EULAR Standing Committee on International Clinical Studies Including Therapeutic Trials (ESCISIT). Rheumatology (Oxford) 2001;40(1):89–94. 11157147 10.1093/rheumatology/40.1.89

[107] Ståhl Hallengren C Nived O Sturfelt G. Outcome of incomplete systemic lupus erythematosus after 10 years. Lupus 2004;13(2):85–8. 14994999 10.1191/0961203304lu477oa

[108] Calvo-Alen J Alarcon GS Burgard SL Burst N Bartolucci AA Williams HJ. Systemic lupus erythematosus: predictors of its occurrence among a cohort of patients with early undifferentiated connective tissue disease: multivariate analyses and identification of risk factors. J Rheumatol 1996;23(3):469–75. 8832985

[109] Danieli MG Fraticelli P Salvi A Gabrielli A Danieli G. Undifferentiated connective tissue disease: natural history and evolution into definite CTD assessed in 84 patients initially diagnosed as early UCTD. Clin Rheumatol 1998;17(3):195–201. 9694051 10.1007/BF01451046

[110] Szodoray P Nakken B Barath S Gaal J Aleksza M Zeher M Progressive divergent shifts in natural and induced T-regulatory cells signify the transition from undifferentiated to definitive connective tissue disease. Int Immunol 2008;20(8):971–9. 18550583 10.1093/intimm/dxn056

[111] Arbuckle MR McClain MT Rubertone MV Scofield RH Dennis GJ James JA Development of Autoantibodies before the Clinical Onset of Systemic Lupus Erythematosus. N Engl J Med 2003;349:1526–33. 14561795 10.1056/NEJMoa021933

[112] Eriksson C Kokkonen H Johansson M Hallmans G Wadell G Rantapää-Dahlqvist S. Autoantibodies predate the onset of systemic lupus erythematosus in northern Sweden. Arthritis Res Ther 2011;13(1):R30. 21342502 10.1186/ar3258 PMC3241374

[113] Eriksson C Rantapää-Dahlqvist S. Cytokines in relation to autoantibodies before onset of symptoms for systemic lupus erythematosus. Lupus 2014;23(7):691–6. 24531079 10.1177/0961203314523869

[114] Md Yusof MY Psarras A El-Sherbiny YM Hensor EMA Dutton K Ul-Hassan S Prediction of autoimmune connective tissue disease in an at-risk cohort: prognostic value of a novel two-score system for interferon status. Ann Rheum Dis 2018;77(10):1432–9. 29929956 10.1136/annrheumdis-2018-213386 PMC6161671

